# “Adjust Zang and arouse spirit” electroacupuncture ameliorates cognitive impairment by reducing endoplasmic reticulum stress in db/db mice

**DOI:** 10.3389/fendo.2023.1185022

**Published:** 2023-04-19

**Authors:** Mengyuan Li, Lin Yao, Min He, Haipeng Huang, Haizhu Zheng, Shiqi Ma, Zhen Zhong, Shuo Yu, Mengmeng Sun, Hongfeng Wang

**Affiliations:** ^1^ Institute of Acupuncture and Massage, Northeast Asian Institute of Traditional Chinese Medicine, Changchun University of Chinese Medicine, Changchun Jilin, China; ^2^ Northeast Asian Institute of Traditional Chinese Medicine, Changchun University of Chinese Medicine, Changchun, China; ^3^ College of Acupuncture and Massage, Changchun University of Chinese Medicine, Changchun Jilin, China

**Keywords:** diabetic cognitive impairment (DCI), endoplasmic reticulum stress (ERS), insulin signaling pathway, hippocampus, neuronal apoptosis

## Abstract

**Introduction:**

Diabetic cognitive impairment (DCI) is a chronic complication of the central nervous system (CNS) caused by diabetes that affects learning and memory capacities over time. Recently, acupuncture has been shown to improve cognitive impairment in streptozotocin-induced diabetic rats. However, the effects of electroacupuncture on DCI and its underlying mechanism have not yet been elucidated in detail.

**Methods:**

In this study, we used db/db mice as DCI animal models which showed low cognitive, learning and memory functions. Electroacupuncture significantly ameliorated DCI, which is reflected by better spatial learning and memory function using behavioral tests. The db/db mice with cognitive impairment were randomly divided into a model group (Mod) and an electroacupuncture treatment group (Acup), while db/m mice were used as a normal control group (Con). First, the mice were subjected to behavioural tests using the Morris water maze (MWM), and body weight, blood glucose, insulin, triglycerides (TG) and total cholesterol (TC) were observed; HE, Nissl, and TUNEL staining were used to observe the morphological changes and neuronal apoptosis in the mice hippocampus; Finally, Western blot and rt-PCR were applied to detect the essential proteins and mRNA of ERS and insulin signalling pathway, as well as the expression levels of Tau and Aβ.

**Results:**

Electroacupuncture significantly ameliorated DCI, which is reflected by better spatial learning and memory function using behavioral tests. Moreover, electroacupuncture attenuated diabetes-induced morphological structure change, neuronal apoptosis in the hippocampus of db/db mice. Our results revealed that electroacupuncture could regulate the expression levels of Tau and Aβ by improving hippocampal ERS levels in db/db mice, inhibiting JNK activation, attenuating IRS1 serine phosphorylation, and restoring normal transduction of the insulin signaling pathway.

**Discussion:**

In summary, ERS and insulin signaling pathway paly causal roles in DCI development. Electroacupuncture can significantly alleviate the pathogenesis of DCI, improve mice's learning and memory ability, and improve cognitive dysfunction. This study adds to our understanding of the effect of acupuncture on DCI and opens the door to further research on DCI.

## Introduction

Diabetes mellitus (DM) is a chronic metabolic disease that may adversely affect several systems. DM may lead to severe neurological lesions in the central and peripheral nervous systems. In particular, damage to the central nervous system (CNS) can cause neurophysiological and structural changes, further leading to cognitive decline (1). Diabetic cognitive impairment (DCI) is one of the chronic complications of diabetes characterized by cognitive impairment, mainly manifested as impaired learning ability, memory function, perception, and central executive function, which is not pertinent to patient’s age and educational level. Consequently, patients with DCI can seriously affect their quality of life and reduce the self-management ability, probably aggravating the progression of diabetes and forming a vicious circle. Therefore, it is of great theoretical significance and social value to carry out in-depth investigation of DCI pathogenesis and explore new targets for the prevention and treatment of DCI.

DCI has a clinicopathological manifestation between normal brain ageing and Alzheimer’s disease (AD), and belongs to the pre-AD stage but does not meet the diagnostic criteria for AD (2). DCI and AD have a similar pathological basis, showing Aβ deposition and Tau hyperphosphorylation (3). Although multiple factors may contribute to DCI, its exact pathogenesis is still unclear. Impaired insulin signaling in the brain, abnormal phosphorylation of Tau protein, glucose toxicity, oxidative stress, endoplasmic reticulum stress (ERS), and amyloid β-protein (Aβ) deposition are reported to be associated with the pathogenesis of DCI (4). Studies have shown that the insulin signaling pathway can regulate the release and clearance of Aβ by allowing β-amyloid precursor protein (APP) to produce secreted APP (sAPP) (5–7). In the case of ERS, it is activated in brain neuronal cells in DCI and AD, and three stress marker molecules on the endoplasmic reticulum are dissociated from glucose-regulated protein 78 (GRP78), of which inositol-requiring enzyme 1α (IRE1α) can activate c-Jun N-terminal kinase (JNK) (3, 8).

As an essential part of traditional medicine, acupuncture has the characteristics of precise efficacy, multi-target, multi-link, and multi-level action. Acupuncture can also effectively improve the cognitive function and daily living ability of patients with cognitive impairment (9–11). Noteworthily, our previous studies have shown that electroacupuncture is very effective in treating diabetes and its complications, regulating blood glucose, improving nerve cell apoptosis, and delaying nerve damage (12). It has been reported that electroacupuncture protects nerve cells by stimulating the electrophysiological characteristics of human meridians and collaterals to strengthen the metabolic circulation of brain blood (13, 14). Since no effective treatment for DCI is available, currently, mainly based on hypoglycemic drugs, a new treatment strategy is urgently needed. On the basis of our previous study and the aetiology of DCI, we propose a new treatment method, “Adjust Zang Arouse Spirit” electroacupuncture”, where Baihui (GV20) and Shenting (GV24) are essential acupuncture points for treating mental disorders (15). This study was designed to prove the effectiveness of the “Adjust Zang Arouse Spirit” electroacupuncture treatment for DCI and to explore its mechanism of action. We focused on ERS, an important link to the pathogenesis of DCI. To understand it in more detail, we explored the effects of electroacupuncture on hippocampal ERS and insulin signaling in mice with DCI. This study is essential to reveal the effects and mechanism of action of “Adjust Zang Arouse Spirit” acupuncture in the treatment of DCI and may offer hope for new therapeutic avenue in DCI.

## Materials and methods

### Mice model of DCI

The T2D-the genetic LepR db/db (db/db) model is an autosomal stealth derivative of an inbred strain of C57BL/KsJ mice with a Leptin receptor site mutation resulting in a Leptin signaling pathway disruption, resulting in obesity, subsequently elevated blood glucose, insulin resistance and fatty liver (16). This phenomenon is most evident in mice from 8-12 weeks, with the development of diabetes-related complications. The db/db mice have spatial learning and memory dysfunction. Therefore, it can be seen as a typical model for studying DCI (17, 18).

A total of 15 male db/m mice (27 ± 3g) and 35 male db/db mice aged 8 weeks (40 ± 4g) were purchased from Changzhou Cavins Laboratory Animals Technology Co. Ltd. (Jiangsu, China; animal license no: SCXK (Su) 2016-0010]. These animals were housed and raised under standard laboratory conditions (21~25°C temperature; 40%~70% humidity; noise < 60 dB; 12:12 h light-dark cycle) at the animal experiment center of Changchun University of Chinese Medicine. The present study was approved by the Ethics Committee of the Changchun University of Chinese Medicine (Approval Number: 2020188). After acclimatization, mice were fed a breeding and growth feed diet (carbohydrate 60%, protein 22%, fat 4.0%), 5 per cage, free feeding and watering. All experimental procedures are strictly in accordance with the Ministry of Science and Technology’s “Guiding Opinions on Treating Experimental Animals Properly” (Version 2006), and the guidelines for the nursing and use of experimental animals of Changchun University of Chinese Medicine.

### Screening for cognitive impairment in db/db mice

All mice were housed adaptively for 7 days prior to the beginning of the experiment. At 9 weeks of age, the Morris water maze (MWM) was used to test the spatial learning and memory abilities of animals in each group to verify the presence of cognitive impairment in db/db mice.

### Animal treatment

The db/db mice with cognitive impairment were randomly divided into model control group (Mod) and electroacupuncture treatment group (Acup). The db/m mice reared under the same conditions were used as the normal control group (Con). At baseline, mice were measured for body weight, blood glucose, and activity, and the results were used to remove abnormal values. During the experiment, all three groups of mice were given the same diet and water. The Acup received electroacupuncture treatment, administered once per day, for 20 min each time, and six treatments were counted as a course of treatment, with continuous treatment for four courses. The main acupuncture points include: Baihui (GV20), Shenting (GV24), and bilateral Feishu (BL13), Pishu (BL20), Shenshu (BL23), Hegu (L14), Zusanli (ST36), Sanyinjiao (SP6), and Taichong (LR3). Acupoints were located according to the atlas of acupuncture points “Experimental Acupuncture and Moxibustion” (Beijing: China Traditional Chinese Medicine Publishing House Co., Ltd, 2021) (19). Mice were treated by a 0.18*13-mm acupuncture needles for single use (Zhongyan Taihe, Beijing Medical Instrument Co., Ltd, Beijing, China). Each acupuncture point is needled in the reverse direction of penetration, the unilateral GV20-GV24, BL20-BL23, and SP6-LR3 were used as electroacupuncture connection pairs by using Huatuo SDZ-V electronic acupuncture therapy instrument (Hwato, Suzhou Medical Appliance Factory, Suzhou, China), taking dilatational wave with frequency of 2 Hz (frequency ratio 1:5). A slight contraction of the muscles determined the intensity of the acupuncture point, and the mice could tolerate it. The duration of electroacupuncture treatment was 20 min. The mice in the Mod and Con group were fixed to the experimental plate in the same way as those in the Acup group, but without treatment, and restrained for 20 min. Throughout the experiment, we made every effort to minimize the pain of animals.

During the 4-week-treatment period, food intake and water intake were measured once a day in each group; body weight and blood glucose were measured at baseline, one week of treatment, two weeks of treatment, three weeks of treatment, and the end of treatment at week 4. At the end of all sessions, the mice in each group underwent MWM again to determine learning and cognitive function. After measuring basic indicators, mice were anaesthetized with breathing isoflurane. For hematoxylin & eosin (H&E), Nissl, and TUNEL staining, the brains were quickly separated, rinsed in saline, then perfused with 4% paraformaldehyde (PFA) in 0.1 M PBS following the saline solution perfusion for 24 h and embedded in paraffin for sectioning. For western blot and polymerase chain reaction, the hippocampus was isolated and stored rapidly at -80°C.

### Morris water maze test

The Morris water maze consists of a cylindrical pool, a columnar station with flexible positioning, an animal behavior trajectory analysis system, and a camera system. The test was performed in a circular pool with a diameter of 120 cm and a height of 50 cm (The platform with a height of 25 cm and a diameter of 8 cm) (Zhongshidichuang Science and Technology Development Co., Ltd., Beijing, China). There are 4 central points on the wall of the pool, N (north), E (east), S (south) and W (west), dividing the pool into four quadrants, I, II, III, and IV. It was filled with clean water, the water depth was about 25 cm, and the water was about 0.5 cm higher than the upper layer of the platform. Water temperature was maintained at 23 ± 0.5 °C using an automatic heating system. MWM records escape latency and the number of the original platform crossings in mice. Each time, the mice were randomly assigned to one of the four quadrants and swam freely to find the platform. If the platform was not found within 60 s, it would be directed there for 15 s, and the escape latency was recorded as 60 s. Mice were trained for 5 days and used to examine spatial learning ability. The escape latency and the swimming trajectories were recorded. A spatial probe trial was performed on day 6 to test the learning and memory abilities of the mice, and the platform was removed. These mice were allowed to swim freely in water without a platform for 60 s. The number of times the mice crossed the platform site, the swimming distance and time in the target quadrant, and the swimming distance and time in the entry quadrant were recorded.

### H&E staining and Nissl staining

The brains were rapidly isolated and placed in a 4% PFA fixative solution for 24 h at room temperature. The brain is then dehydrated in alcohol and embedded in paraffin. The 3-µm sections were dewaxed and hydrated, then stained with hematoxylin and eosin solution (Servicebio Science and Technology, Beijing, China). Morphological changes in brain tissue were observed under light microscopy. For Nissl staining, tissue sections were stained with cresol violet. They were differentiated using Nissl differentiation solution (Servicebio Science and Technology, Beijing, China) and dehydrated, transparent, and sealed. Neuronal staining in the hippocampal region of brain tissue was observed under light microscopy. Neuronal staining in the hippocampal region of brain tissue was observed under light microscopy, and the number of Nissl bodies was determined using Image-Pro plus software.

### Enzyme-linked immunosorbent assay (ELISA)

The blood was obtained from the eyes of anaesthetized mice and transferred to anticoagulation tubes. After 2 hours at 4°C, the blood samples were centrifuged at 4000 rpm for 10 minutes, then we collected serum, and measured the levels of total cholesterol (TC) (Jiangsu Cote Biological Technology Co. Ltd., Product Number: KT30043-B), triglyceride (TG) (Jiangsu Cote Biological Technology Co. Ltd., Product Number: KT30053-B), and insulin (INS) (Jiangsu Cote Biological Technology Co. Ltd., Product Number: KT2579-B) using an ELISA kit according to the manufacturer’s instructions.

### Western blot analysis

For protein extraction, the hippocampus was homogenized in lysis buffer containing protease inhibitor cocktail (Beijing Labgic Technology Co., Ltd., Product No. BL539A). Then, the complexes were centrifuged at 12,000 rpm for 10 min at 4°C, and the supernatant obtained was used for protein determination. The Bradford technique was used to determine the protein concentration. The isolated proteins were quantified using BCA assay. Separation of 20 ug protein by SDS-PAGE (8, 10, 12 or 15% gel) and transfer to PVDF membrane. After being blocked with 5% skimmed milk for 0.5 h at room temperature, the membranes were incubated overnight at 4°C with the specific primary antibodies. After washing 3 times with TBST (TrisHCL (1M, pH7.5): 50mL; Nacl: 8g; KCL: 0.2g; Tween: 0.5ml), the membranes were treated with a secondary antibody of the same genus for 2 hours at room temperature. ECL chemiluminescence detection reagents were used and detected using a gel imaging analyzer. The Alpha software (Alpha Innotech, San Leandro, CA, USA) processing system was used to analyse the optical density values of the target band. All experiments were repeated in triplicate with independently prepared tissue.

### Quantitative real-time polymerase chain reaction (rt-PCR)

Total RNA was extracted from hippocampus tissues using Trizol reagent and transcribed to cDNA using the Servicebio^®^RT First Strand cDNA Synthesis Kit (Wuhan servicebio technology Co., Ltd., Product No. G3330) according to the protocol of our laboratory. The total RNAs were quantified by spectrophotometer (Bio-Rad, Hercules, CFX). cDNA was amplified for 5 min at 25°C, 30 min at 42 °C, and the terminative reaction temperature was 85°C for 5min. The change in cRNA expression was calculated employing the comparative change-in-cycle method (ΔΔCt), with GAPDH as the standardized gene.

### TUNEL staining

TUNEL staining was performed using an in Situ Cell Death Assay kit (Wuhan servicebio technology Co., Ltd., Product No. G2006) to determine apoptosis in the hippocampal region by TdT-mediated dUTP nick-end labeling according to the manufacturer’s instructions. After dewaxingand hydration, the brain sections was incubated with proteinase K working solution for 20 min at 37°C. The slides were then rinsed 3 times with phosphate buffer saline (PBS, hyclone, America Co., Ltd., Product No. SH30256.01), which was followed by incubation with the TUNEL reaction mixture. Finally, use the DAPI dye solution to seal for 5 min at room temperature. TUNEL-positive and total cells were observed under a fluorescent microscope (Nikon Corporation, Eclipse Ci-L, Japan). TUNEL-positive cells and neurons were expressed as a percentage of total cells.

### Statistical analysis

All the data were processed using SPSS 26.0 statistical analysis software and were expressed as mean ± SEM. If the experimental data conform to the normal distribution and meet the variance homogeneity test, the one-way analysis of variance (ANOVA) was used. In one-way ANOVA analysis, the Tukey test was used to estimate the significance of the results (p<0.05). Statistical results were considered significantly different at p<0.05.

## Results

### Electroacupuncture treatment improved the spatial learning and memory abilities of db/db mice

**Figure 1:**
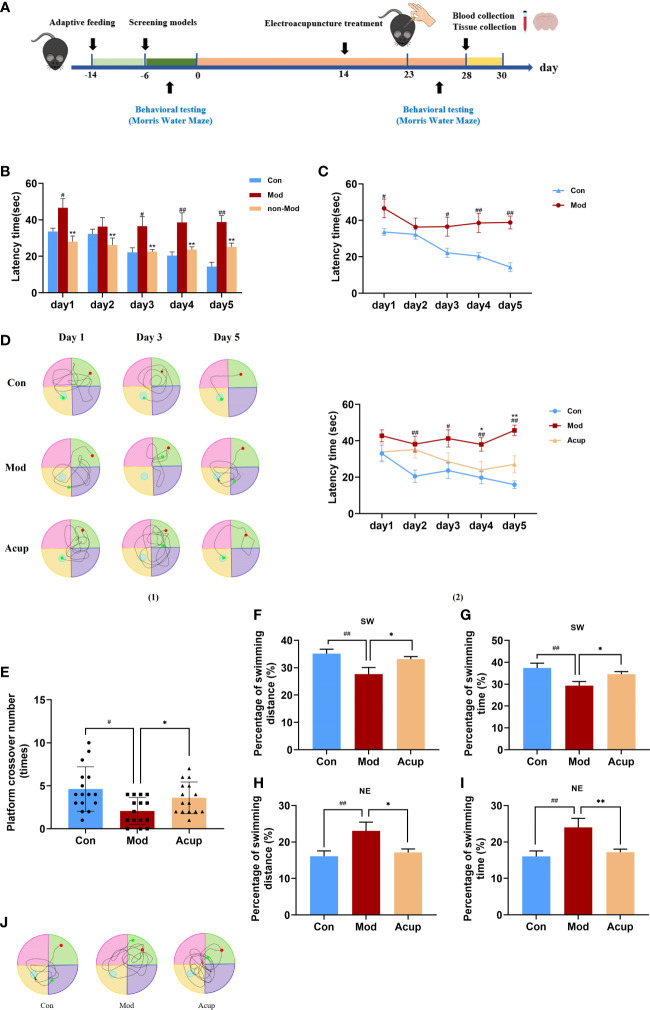
Electroacupuncture improves db/db mice’s learning and memory abilities of the MWM test, respectively. **(A)** Timeline of experimental procedures. **(B, C)** A comparison of escape latency in each group from day 1 to day 5 before treatment. **(D)** A comparison of escape latency in each group from day 1 to day 5 after treatment. **(E)** A comparison of passing times in each group through the platform quadrant on the sixth day. **(F)** A comparison of percentage of swimming distance in each group through the platform quadrant (SW) on the sixth day. **(G)** A comparison of percentage of swimming time in each group through the platform quadrant (SW) on the sixth day. **(H)** A comparison of percentage of swimming distance in each group through the entry quadrant (NE) on the sixth day. **(I)** A comparison of percentage of swimming time in each group through the entry quadrant (NE) on the sixth day. **(J)** The swimming track in each group on the sixth day in 60 s. Non-Mod represents db/db mice model without cognitive impairment. Data are expressed as means ± SEM. Mod vs Con, ^#^ *p* < 0.05, ^##^ *p* < 0.01; Acup vs Mod, ^*^ *p* < 0.05, ^**^ *p* < 0.01.

MWM was used to detect the spatial learning and memory ability of db/db mice. Before the beginning of the experiment, all mice underwent MWM behavior tests for six days to determine whether db/db mice had cognitive impairment (**Figure 1A**). After testing, it was found that compared with the Con group, db/db mice in the Mod group showed worse learning and spatial memory ability (p<0.05 or p<0.01) (**Figures 1B, C**). It indicated that db/db mice had cognitive impairment, which proved that the model was established. However, not all db/db mice have cognitive impairment, five mice (non-Mod) were excluded after MWM screening. The mice were trained to locate the platform, and all mice struggled to swim to the platform. In each group, the escape latency of mice decreased with increased training time. There was a significant difference in the escape latency in the Acup group to reach the platform on the fourth day of training compared with that in the Mod group (p<0.05), bringing it closer to that in the Con group (**Figure 1D** (1)- (2)). These results showed that electroacupuncture could improve the spatial learning ability of db/db mice.

On the sixth day of testing, we removed the platform and tested the difference in the spatial memory ability of the mice in a spatial probe trial. When the platform was removed, we tested the number of times that the mice crossed the platform, the swimming distance and time in the target quadrant, the swimming distance and time in the entry quadrant, and the swimming track within 60 s. Swimming distance was positively correlated with escape latency. Fewer crossings over the platform position was observed in the Mod group than those in theAcup group (p<0.05) (**Figure 1E**). In addition, as shown in **Figures 1F–I**, the mice in the Acup group and Mod group showed similar escape latency in percentage of swimming time and swimming distance in target and entry quadrants. The swimming track of mice in each group had a particular tendency, the mice in the Con group and Acup group swam in the target quadrant, while those in the Mod group swam in the entry quadrant, with the longest and most complex swimming track (**Figure 1J**). The above results showed that electroacupuncture treatment brought the spatial learning ability of the mice closer to the level of the Con group. It is suggested that electroacupuncture may have beneficial effects on the spatial learning and memory abilities of db/db mice.

### Electroacupuncture treatment alleviated DCI-related physiological indicators in db/db mice

**Figure 2:**
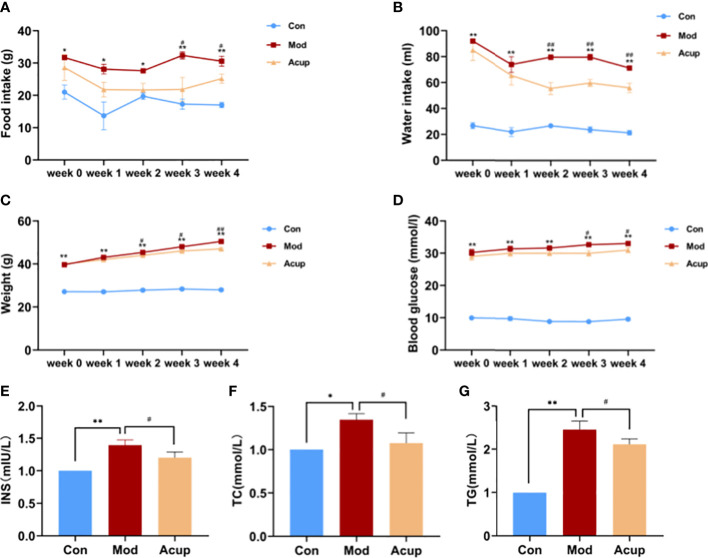
Effects of electroacupuncture on general indicators in db/db mice. **(A)** Food intake, **(B)** Water intake, **(C)** Weight and **(D)** Blood glucose changes before and after electroacupuncture treatment. **(E)** INS, **(F)** TC and **(G)** TG levels in mice serum were analyzed using Elisa kit. Data are expressed as means ± SEM. Mod vs Con, ^#^ *p* < 0.05, ^##^ *p* < 0.01; Acup vs Mod, ^*^ *p* < 0.05, ^**^ *p* < 0.01.

In order to evaluate the effects of “Adjust Zang and Arouse Spirit” electroacupuncture on db/db mice, we monitored the food intake, water intake, body weight, blood glucose, and some other diabetes-related serological indicators in each group. Before treatment (week 0), the values of food intake and water intake of mice in the Mod group and the Acup group were significantly higher than those in the Con group, and there was no significant difference between the Mod and Acup groups. However, compared with the Mod group, the food intake and water intake were seen reduced significantly in the Acup group after 3 and 2 weeks of electroacupuncture treatment (**Figures 2A, B**). At the same time, in comparison, the body weight was significantly improved in the Acup group after 2-week intervention, so as the blood glucose was also reduced after 3-week treatment (**Figures 2C, D**). In order to determine the effect of electroacupuncture treatment on IR and evaluate the function of islet β cells, insulin (INS) was also calculated in mice serum. The result showed INS in the Acup group was significantly reduced in comparison with that in the Mod group (**Figure 2E**).

Furthermore, electroacupuncture treatment significantly inhibited the increase of total cholesterol (TC) and triglyceride (TG) levels in serum in the Acup group compared to those in the Mod group (**Figures 2F, G**). According to the above results, it is suggested that “Adjust Zang and Arouse Spirit” electroacupuncture can improve the common indicators in db/db mice.

### Electroacupuncture treatment modulated morphological structure changes and neuronal apoptosis in hippocampus of db/db mice

**Figure 3:**
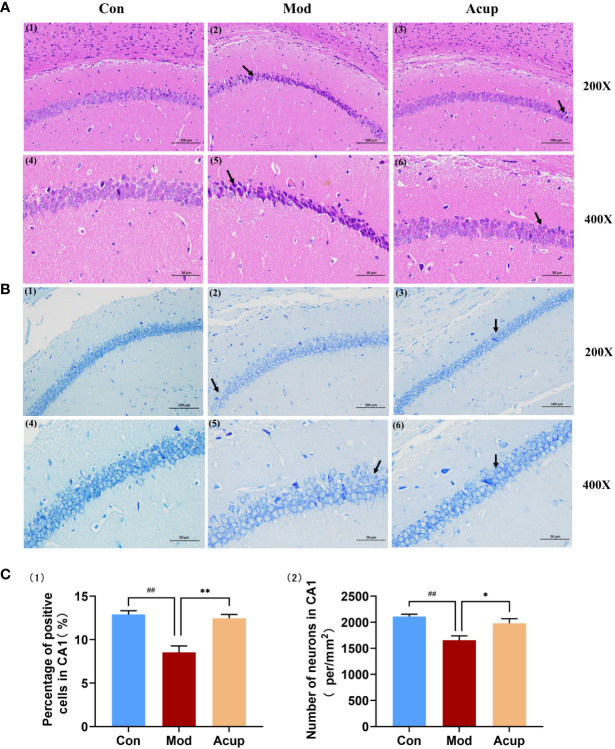
Electroacupuncture regulates morphological changes in the hippocampus of db/db mice. **(A)** The representative images of H&E stained mouse hippocampal sections in the three groups (20×/200×/400×). **(B)** The representative images of Nissl staining of the mouse hippocampal sections in the three groups (20×/200×/400×). **(C)** (1) Percentage of positive cells in the CA1 region of the mouse hippocampus; (2) Number of neurons per unit area in the CA1 region of the db/db mice’s hippocampus. Data are expressed as means ± SEM. Mod vs Con, ^##^ *p* < 0.01; Acup vs Mod, ^*^ *p* < 0.05, ^**^ *p* < 0.01.

The hippocampus plays an essential role in learning and memory and is thought to be a specific target for diabetes-related changes (20). In this study, the neurons in the hippocampal CA1 region of the mice in the Mod group were widely lost, with nuclear rupture, nuclear pyknosis, and sparse arrangement (**Figure 3A**(1)-(6)). The number of neurons in the hippocampal CA1 region of the Acup group was increased, the staining was evident, and the number of Nissl bodies in the cytoplasm was significantly increased compared with those in the Mod group (**Figure 3B**(1)-(6)). The percentage of positive cell area and the number of neurons per unit area in the hippocampal CA1 region were also significantly increased in the Acup group compared to those in the Mod group (p<0.05, p<0.01) (**Figure 3C**(1)-(2)). It is suggested that electroacupuncture intervention may repair damaged neurons in the hippocampus of db/db mice and exert a neuro-protective effect.

**Figure 4:**
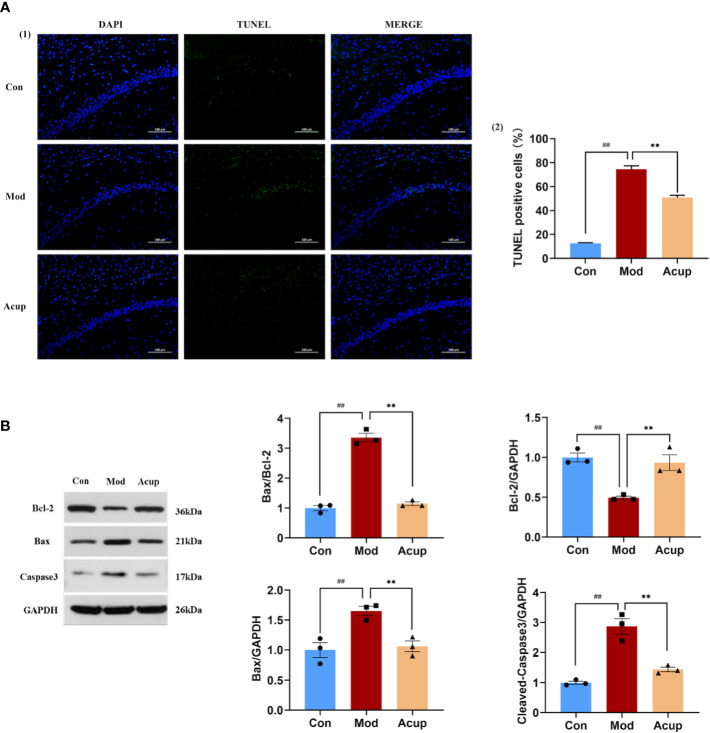
Electroacupuncture regulates db/db mice’s neuronal apoptosis in the hippocampus. **(A)** (1) The representative image of TUNEL assay showing apoptotic cells (green signal) in the CA1 regionof the db/db mice’s hippocampus. (2) The proportion of apoptotic neurons in the CA1 region of the hippocampus in three groups. **(B)** Western blotting and quantitative analysis of Bax, Bcl-2, and caspase-3 expression in the hippocampus of mice in three groups. Data are expressed as means ± SEM. Mod vs Con, ^##^ *p* < 0.01; Acup vs Mod, ^**^ *p* < 0.01.

The results of the TUNEL assay also showed that the proportion of apoptotic neuronal cells in the CA1 region of the hippocampus was inhibited in the Acup group compared to that in the Mod group (p<0.01) (**Figure 4A**(1)-(2)). In contrast, WB analysis showed that Bcl-2 expression was reduced, and Bax expression was increased in the hippocampus of db/db mice in the Mod group (p<0.01) (**Figure 4B**). More importantly, the split level of caspase-3 was significantly higher in the hippocampus of db/db mice compared to that in Acup group (p<0.01) (**Figure 4B**), revealing that electroacupuncture treatment can reverse these results. These results indicate that electroacupuncture treatment can effectively improve the degree of damage to hippocampal neurons in db/db mice and protect the neuronal cell morphology *via* restoring the normal function of neurons.

### Electroacupuncture intervention regulated the expression of essential proteins related to the ERS in db/db mice

**Figure 5:**
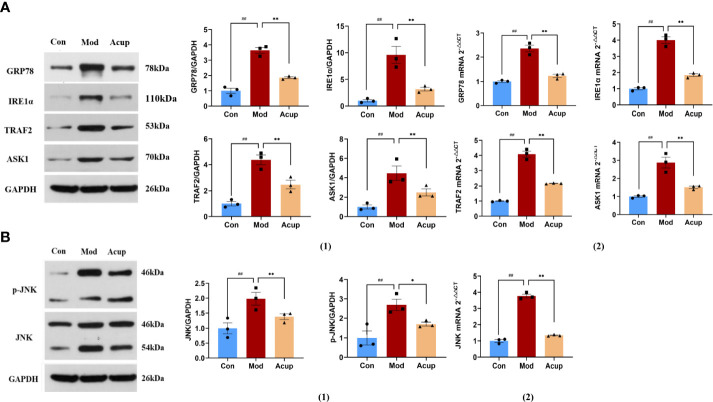
Electroacupuncture regulates the expression of key proteins in the ERS. **(A)** (1) Western blotting and quantitative analysis of GRP78, IRE1α, TRAF2 andASK1 expression in the hippocampus of mice in three groups; (2) RT-PCR analysis of GRP78, IRE1α, TRAF2 and ASK1 mRNA expression in the hippocampus of mice in three groups. **(B)** (1) Western blotting and quantitative analysis of JNK and p-JNK expression in the hippocampus of mice in three groups; (2) RT-PCR analysis of JNK mRNA expression in the hippocampus of mice in three groups. Data are expressed as means ± SEM. Mod vs Con, ^##^ *p* < 0.01; Acup vs Mod, ^*^ *p* < 0.05, ^**^ *p* < 0.01.

Many studies have suggested that ERS plays a vital role in the various pathological aspects of DCI development (21–23). In our research, WB and PCR were used to detect the expression of essential proteins related to the ERS and insulin signaling pathways in db/db mouse hippocampus. We examined the expression of ERS markers in the hippocampus and found that the protein and mRNA levels of GRP78, IRE1α, TNF receptor-associated factors 2 (TRAF2) and apoptosis signal-regulating kinase 1 (ASK1) were significantly decreased in the mouse hippocampal tissues in the Acup group when compared with those in the Mod group (p<0.05 or p<0.01) (**Figure 5A**(1)-(2)). Additionally, the expression levels of c-Jun N-terminal kinase (JNK) and p-JNK were decreased in the Acup group in comparison to those in the Mod group (p<0.05) (**Figure 5B**(1)-(2)). Here, we verified that electroacupuncture intervention affected Aβ and Tau expression by activating the IRE1α-JNK signaling pathway, initiating JNK-dependent IRS1 to phosphorylate it and thereby inhibiting the insulin signaling pathway.

### Electroacupuncture treatment affected the expression of essential proteins related to the insulin signaling pathway

**Figure 6:**
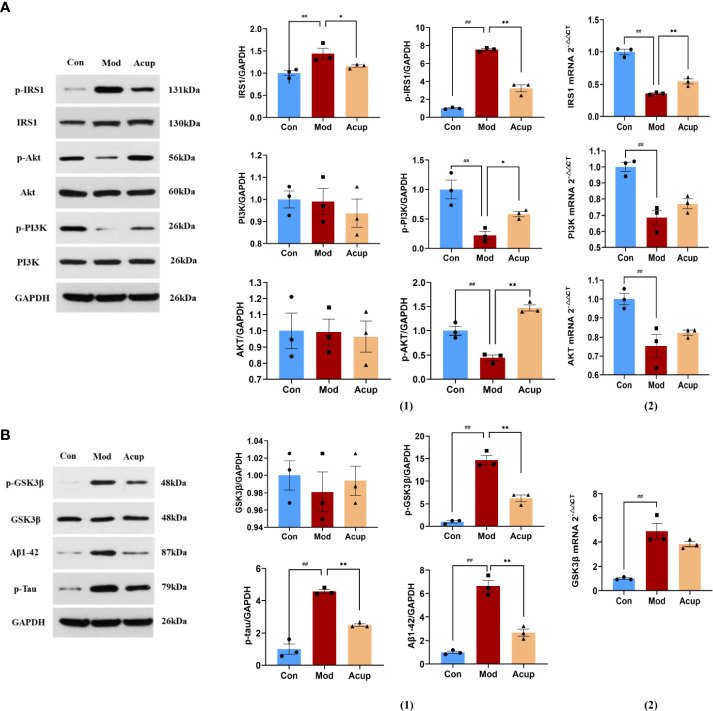
Electroacupuncture regulates the expression of key proteins of the insulin signaling pathway. **(A)** (1) Western blotting and quantitative analysis of IRS1, p-IRS1, PI3K, p-PI3K, AKT, and p-AKT expression in the hippocampus of mice in the three groups; (2) RT-PCR analysis of IRS1, PI3K and AKT mRNA expression in the hippocampus of mice in the three groups. **(B)** (1) Western blotting and quantitative analysis of GSK3β, p-GSK3β, Aβ and Tau expression in the hippocampus of mice in the three groups; (2) RT-PCR analysis of GSK3β mRNA expression in the hippocampus of mice in the three groups. Data are expressed as means ± SEM. Mod vs Con, ^##^ *p* < 0.01; Acup vs Mod, ^*^ *p* < 0.05, ^**^ *p* < 0.01.

PI3K and Akt are essential downstream molecules that reflect the normal transduction of the insulin signaling pathway. When the insulin signaling pathway is abnormal, the phosphorylation expression of PI3K and AKT is significantly reduced (24). In this study, we detected the expression of Insulin receptor substrate 1 (IRS1), p-IRS1, phosphatidylinositol-3-hydroxykinase (PI3K), p-PI3K, protein kinase B (AKT), and p-AKT in the mouse hippocampus. Our findings indicated that there was no significant difference in protein and mRNA expression of PI3K and AKT between the Mod and the Acup groups. Meanwhile, when compared to the Mod group, the Con group and Acup group had significantly lower IRS1 and p-IRS1 levels (p<0.05 or p<0.01), higher p-PI3K and p-AKT levels (p<0.05 or p<0.01) (**Figure 6A**(1)-(2)), showing that acupuncture may have a regulatory influence on the levels of key proteins in insulin signaling pathway in the db/db mice.

When insulin signal transduction was impaired, AKT lost its inhibition of glycogen synthase kinase-3β (GSK3β), increased the expression of Aβ and Tau, and further aggravated cognitive impairment. According to our findings, the Acup group’s expression of p-GSK3β, Aβ and Tau was lower as compared to the Mod group, but there was no substantial difference in the GSK3β between the two groups (p<0.01) (**Figure 6B**(1)-(2)). These findings suggest that acupuncture can influence the expression of Aβ and tau proteins in db/db mice by regulating key downstream proteins of insulin signaling.

## Discussion

DCI is a severe, chronic diabetic-induced central nervous system complication that can lead to changes in brain structure and function (25). The pathological mechanisms of DCI are extremely complex. Currently, there is no effective drug to treat diabetes-related cognitive impairment. Electroacupuncture shows good therapeutic effect on cognitive impairment (26). Our results reveal that electroacupuncture can improve the learning and memory function of diabetic mice by reducing ERS, inhibiting JNK expression and removing Aβ and Tau from hippocampal neuron cells.

In this study, the “Adjust Zang-fu and Arouse Spirit” electroacupuncture treatment method has been chosen to treat cognitive impairment caused by diabetes, and this acupuncture method uses BL13, BL20, BL23, GV20 and GV24 as the main points, supplemented with L14, ST36, SP6 and LR3. BL13, BL20, and BL23 are the back-shu points of the bladder meridian.The back-shu points are the reaction points of the internal organs’ meridian qi in the back, so the BL13, BL20, and BL23 can benefit the lung, spleen, and kidney. GV20 and GV24 are located on the governor meridian. GV20 and GV24, located deep in the brain’s frontal lobe, regulate the nervous system and improve learning and memory. They are both essential acupoints for treating mental illness. ST36 is on the stomach meridian of foot-yang ming, an important point for health care, which has the effect of activating meridians, regulating qi and blood and can improve cognitive function. SP6 is a point where the Foot three Yin Meridian meet, and can tonify the qi and blood of the three meridians of the liver, spleen, and kidney, which has the impact of nourishing the mind, opening the orifices and promoting awakening. The combination of L14 and LR3 has the effect of regulating qi and blood as well as activating the meridians and cognitive-related areas (27). We selected three groups of acupoints, GV20-GV24, BL20-BL23, and SP6-LR3 for electroacupuncture stimulation. Among them, GV20-GV24 can invigorate yang qi, awaken the mind, calm the mind, and enhance intelligence, which may alleviate cognitive impairment caused by DCI. BL20-BL23 can strengthen the spleen, benefit the kidney, nourish the brain and generate marrow. SP6-LR3 can co-regulate yin and yang, tonify qi and invigorate blood. These acupoints are matched together.to achieve the function of regulating the qi and blood of the internal organs, opening the body, and awakening the mind, which may prevent and treat DCI.

In this study, we chose db/db mice as the experimental animal model of DCI and confirmed that db/db mice had cognitive dysfunction through MWM in advance. Compared with the Con group, the spatial learning and memory ability of db/db mice decreased (**Figures 1B, C**). Therefore, db/db mice can be used as a model for DCI research, which is consistent with previous research results (28). In the formal MWM experiment, the performance of the mice after five hidden platforms and one space exploration experiment indicated that acupuncture had a specific promotion role in the space learning and memory ability of db/db mice (**Figures 1D, E**). Swimming distance is related to the escape latency time. In this experiment, mice showed a certain tendency in swimming time and distance in the target quadrant and the entry quadrant. The results showed that acupuncture could induce mice to learn the best swimming style faster and tend to swim more in the target quadrant, resulting in longer swimming time and distance (**Figures 1F–J**). In the MWM-based spatial learning memory test, all values in the Acup group were significantly different compared to those in the Mod group, indicating that acupuncture positively modulated the memory ability of db/db mice.

Currently, there are many animal models of diabetes, including single-gene obesity models, multi-gene obesity models, induced obesity models, non-obesity models, β-cell dysfunction and gene-induced types (29, 30). The choice of each model has its advantages and disadvantages, so it is essential to choose the appropriate animal model for experiments. The animal model selected in this study was spontaneous T2DM db/db mice. After six weeks, they showed apparent obesity, increased fasting blood glucose and insulin levels, and increased water intake and food intake. It is most apparent at 8-12 weeks, diabetic complications can occur. After “Adjust Zang and Arouse Spirit” electroacupuncture intervention, the mice showed positive improvements in body weight, blood glucose, food intake and water intake (**Figures 2A, C**).

DCI is a pathology characterized by learning memory and cognitive impairment in the pathogenesis of DM, with blood glucose instability being a central factor in its development (31). Insulin is the only peptide hormone secreted by pancreatic β-cells that lowers blood glucose and plays a vital role in the body. Insulin connects with the receptor to transport glucose from the cell membrane to the cytoplasm to regulate glucose absorption (32). Although the human brain cannot store glucose, it can enter the brain through the peripheral blood. Thus, when the glucose content in the blood is abnormal, it will inevitably damage brain function. There is a large amount of insulin in different brain areas of humans and animals, especially in learning and memory-related hippocampal areas and prefrontal cortex areas, indicating that insulin may have a specific impact on cognitive functions besides maintaining a blood glucose steady-state (33). Insulin modulates higher brain functions, such as learning and memory, by affecting hippocampal synaptic plasticity (34). In this experiment, in addition to the increase in blood glucose, insulin content in db/db mice also increased significantly. After acupuncture intervention, the levels of blood glucose and insulin significantly declined (**Figures 2D, E**). We speculate that the “Adjust Zang-fu and Arouse Spirit” electroacupuncture may regulate blood glucose by lowering insulin levels and improving insulin resistance. Furthermore, after acupuncture intervention, TC and TG as the blood indicators related to this disease were also reduced considerably (**Figures 2F, G**).

The hippocampus is a complex and essential functional area located in the deep temporal lobe of the brain. The structural and functional integrity of the hippocampus is closely linked to the regulation of short-term memory, learning, executive ability and attention, and cognitive impairment (35–37). DM disrupts the structure and function of neurons, axons and synapses in the CA1 region, affecting synaptic plasticity and long-term potentiation (LTP). Significant hippocampal atrophy in patients aged 60-90 with T2DM has been found in clinical studies, mainly in bilateral hippocampal CA1 area volume reduction and with severe IR (38). Importantly, acupuncture can increase the expression of neurons in the hippocampal CA1 region of mice and restore the number of Nissl bodies; it can also delay the damage and atrophy of the hippocampus (**Figure 3A**(1)-(6))(**Figure 3B**(1)-(6))(**Figure 3C**(1)-(2)).

Apoptosis plays an essential role in diabetes-induced neuronal defects in the hippocampus, mainly in CA1 and CA2 regions of the hippocampus (39, 40). Many factors are involved in this process, and the key components include two protein families, caspases and Bcl-2 (41). Caspase 3 is the essential member of the caspase family and usually exists as an inactive pro-caspase 3 in various tissues and cells. Caspase 3 plays an influential role in neuronal apoptosis in the central nervous system, known as the “killer protein”, and is the final executor of apoptosis. Studies have shown that the hippocampus is a susceptible area of apoptosis in DM mice, and the expression of caspase 3 in the hippocampus is increased (42). Bcl-2 and Bax are essential molecules in ERS-induced apoptosis (43). Bcl-2, widely distributed in the cerebral cortex and hippocampus, is a recognized anti-apoptotic gene (44), while Bax can accelerate the process of cell apoptosis. Relevant studies have shown that in the dentate gyrus of DM mice with cognitive impairment, the expression levels of the Bcl-2 gene and protein are down-regulated, while the expression of Bax is up-regulated, resulting in a significant increase in the ratio of Bax/Bcl-2 (45). Our study showed that the expressions of Bax and caspase 3 in the hippocampus of db/db mice were increased. At the same time, Bcl-2 was decreased, resulting in the loss of neurons and destruction of synaptic structure, while acupuncture could reverse the related changes (**Figure 4A**(1)-(2)) (**Figure 4B**).

Cellular stress due to hyperglycemia is a major mediator in developing T2DM-related complications (46). Several studies have shown that ERS is one of the precipitating factors leading to the development of T2DM-related neuropathy (23, 47). In this study, we found that the activity of IRE1α in the hippocampus of db/db mice increased, and IRE1α was the primary medium of ER-related apoptosis. When ERS occurs, GRP78 dissociates from IRE1α. The IRE1α protein oligomerizes and activates its endoribonuclease activity by autophosphorylation. The enzyme domain of its cytoplasm recruited linker molecules TRAF2 and ASK1 together to form the apoptosis-promoting complex IRE1α-TRAF2-ASK1, which then activated JNK to phosphorylate and trigger cell apoptosis. After acupuncture treatment, the expression levels of ERS marker proteins GRP78, IRE1α, TRAF2, and ASK1, as well as their mRNA transcription levels, were down-regulated (**Figure 5A**(1)-(2)), and the expression of p-JNK in hippocampal neurons was decreased (**Figure 5B**(1)-(2)). This suggests that acupuncture may play an anti-apoptotic role by interfering with ERS-mediated JNK activation.

**Figure 7:**
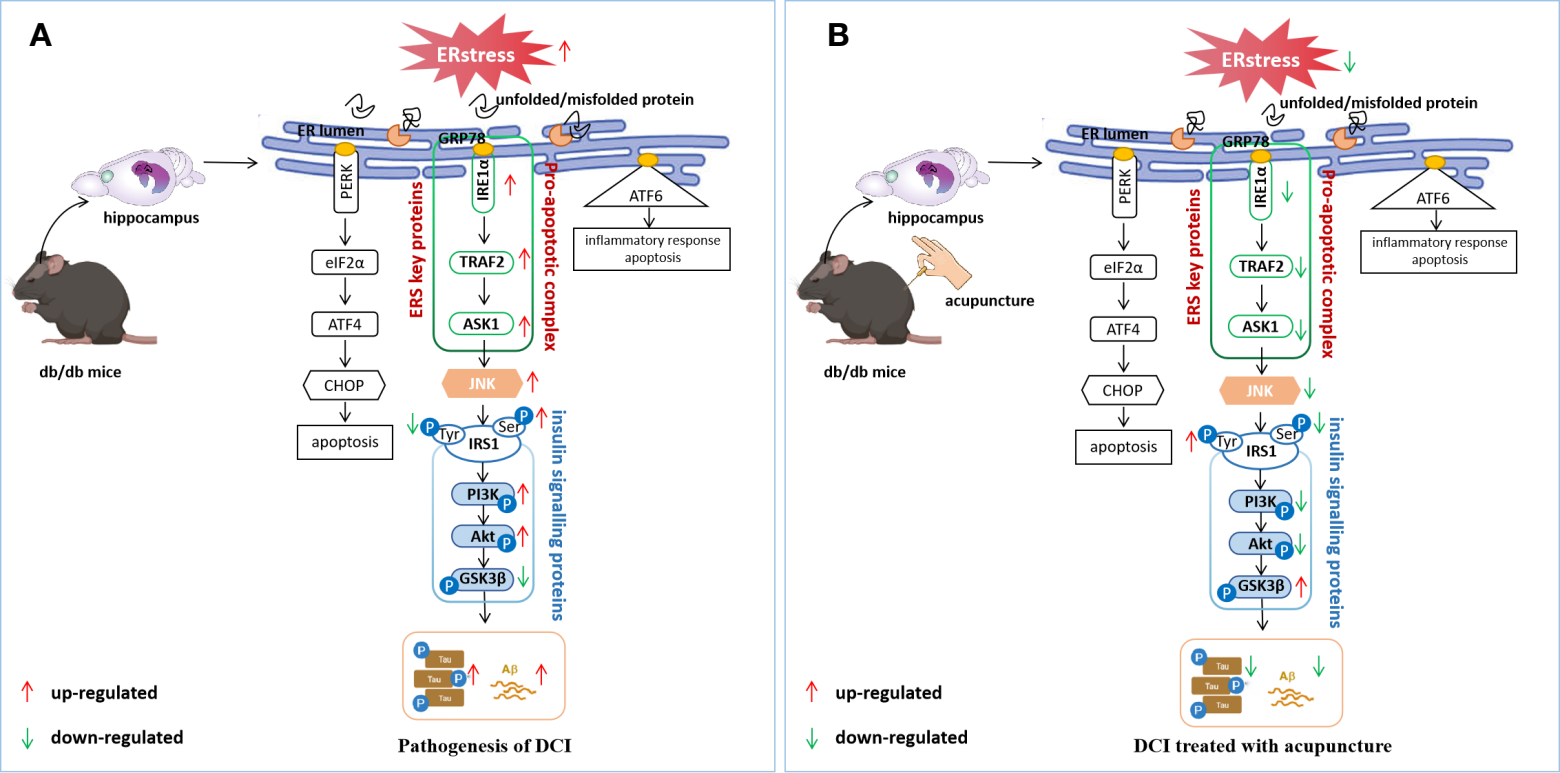
Potential regulatory mechanisms of DCI by acupuncture. **(A)** ERS induces an unfolded protein response that dissociates GRP78 from IRE1α and activates JNK, leading to abnormal insulin signalling pathway transduction, increasing the expression of p-tau and Aβ; **(B)** Electroacupuncture improves cognitive impairment by regulating hippocampal ERS levels in db/db mice, inhibiting JNK activation, restoring normal insulin signalling pathway transduction, and causing a reduction in p-Tau and Aβ expression.

In a diabetic state, ERS leads to JNK activation and initiates JNK-dependent IRS1 serine phosphorylation, affecting insulin signal transduction (48). JNK has been shown to play a vital role in the development of IR (49). In peripheral tissues, ERS mediated by various factors can inhibit insulin signal transduction through JNK phosphorylation, leading to IR. Another aspect that cognitive impairment is closely related to T2DM is the IR of the central nervous system. IR leads to decreased insulin receptor sensitivity and abnormal insulin signal transduction, which affect the expression of Aβ and Tau and interfere with neuronal cell degeneration and cognitive brain function (50). Therefore, this study focused on the perspective of abnormal insulin signaling pathway transduction caused by central IR. As mentioned above, insulin is a major glucose regulator and widely distributed in different brain tissues and involved in the regulation of cognitive function (51). After combining with its receptor, insulin activates its tyrosine activity through the receptor’s phosphorylation, which activates IRS1 and initiates the downstream insulin signaling pathway. Under the condition of IR, phosphorylation of the serine site of IRS1 leads to inhibition of the downstream PI3K-Akt signaling pathway and activation of GSK3β (52). GSK3β is a major Tau protein kinase involved in Tau’s pathological development in AD. Aberrant GSK3β expression leads to Tau hyperphosphorylation and Aβ toxicity (53). In this study, we found that p-IRS1 expression was increased in the hippocampus of db/db mice; expression levels of its downstream molecules p-PI3k and p-Akt were decreased, while p-GSK3β showed the opposite trend. Acupuncture treatment can reverse the abnormal expression of the above molecules, alleviate IR, and restore normal insulin signal transduction (**Figures 6A, B**). Moreover, in this study, we found that Aβ and p-Tau in the hippocampus of mice in the Mod were significantly higher than those in Con. These results confirm that brain IR can lead to cognitive impairment by interfering with Aβ and p-Tau, which is consistent with the results of other researchers (54, 55), Acupuncture can effectively regulate the level of ERS and reduce the expression of Aβ and Tau (**Figures 7A, B**).

## Conclusion

In this study, we demonstrated that insulin signal transduction was impaired in the hippocampus of db/db mice and was closely related to JNK activation induced by ERS activation. “Adjust Zang-fu and Arouse Spirit” electroacupuncture could inhibit JNK activation by reducing ERS, thereby reducing serine phosphorylation of IRS1 and restoring the normal function of insulin signal transduction. It also reduced the expression of p-Tau and Aβ, improved the learning and memory ability of mice, and exerted an effect of improving cognitive function. Therefore, acupuncture, as a holistic therapy, has the potential of significant contribution to the treatment and control of DCI.

## Data availability statement

The original contributions presented in the study are included in the article/**Supplementary Material**. Further inquiries can be directed to the corresponding authors.

## Ethics statement

The animal study was reviewed and approved by the Ethics Committee of the Changchun University of Chinese Medicine.

## Author contributions

ML, LY, HZ, SY, SM and ZZ performed experiments, did the statistical analyses, and participated in data interpretation. ML, LY, HH and MH analyzed data, did the statistical analyses, and participated in data interpretation. MS and HW conceived and designed experiments. ML wrote the article. All authors contributed to the article and approved the submitted version.
