# Supplementary material for: “Adjust Zang and arouse spirit” electroacupuncture ameliorates cognitive impairment by reducing endoplasmic reticulum stress in db/db mice
Source: Front Endocrinol (Lausanne). 2023 Apr 19;14:1185022. doi: 10.3389/fendo.2023.1185022 (PMC10154981; doi:10.3389/fendo.2023.1185022)
Supplement: Supplementary file 1 [file Table_1.docx]

**S1. Anatomical localization of the acupoints mentioned in the article**

| Acupoints | Locations |
| --- | --- |
| Baihui(GV20) | It is located 5 cun straight above the anterior hairline |
| Shenting (GV24) | It is located 0.5 cun straight above the anterior hairline |
| Feishu (BL13) | It is located under the spinous process of the third thoracic vertebra and 1.5 cun apart from the posterior midline |
| Pishu (BL20) | It is located 1.5 cun from the anterior midline of the back |
| Shenshu (BL23) | It is located 1.5 cun at the subspinous depression of the second lumbar spine |
| Hegu (L14) | It is located between the first and second metacarpal bones, when the midpoint of the radial side of the second metacarpal bone |
| Zusanli (ST36) | On the medial side of the lower leg, when the tip of the inner ankle of the foot is 3 cun above, behind the medial edge of the tibia |
| Sanyinjiao (SP6) | It is located on the inside of the calf, 3 cun above the tip of the medial malleolus of the foot, and behind the medial border of the tibia |
| Taichong (LR3) | It is located on the dorsum of the foot, between the first and second metatarsal bones, in the depression anterior to the metatarsal junction, or at the point where the arterial fluctuation is palpable |

Note: "cun" is the traditional Chinese unit of length, 1 cun = 3.33
